# Auricular cartilage tissue engineering: from making cartilage to regenerating a shape-stable elastic organ

**DOI:** 10.3389/fbioe.2026.1835403

**Published:** 2026-08-19

**Authors:** Hongyu Zhang, Jingwei Feng, Jiajun Zhi, Yiwen Deng, Tianqi Yu, Haiyue Jiang, Jiaxian Lin

**Affiliations:** 1 Plastic Surgery Hospital, Chinese Academy of Medical Sciences and Peking Union Medical College, Beijing, China; 2 The Affiliated Nanhua Hospital (Nanhua Clinical College), University of South China, Hengyang, Hunan, China

**Keywords:** 3D printing, auricular cartilage, chondrocytes, elastic cartilage, microtia, regenerative medicine, scaffold, tissue engineering

## Abstract

Auricular cartilage tissue engineering has long been grouped under the broader category of craniofacial cartilage reconstruction, but this classification is becoming insufficient. Unlike articular cartilage repair, which mainly aims to restore load-bearing function and relieve pain, auricular reconstruction must reproduce a complex three-dimensional structure, preserve elastic recoil, resist long-term contraction, tolerate soft-tissue coverage, and remain surgically and aesthetically acceptable over time. These demands make auricular cartilage engineering fundamentally distinct from generic cartilage engineering. Over the past decade, the field has progressed from proof-of-concept ear-shaped constructs in small-animal models to more advanced strategies, including patient-specific scaffold design, expansion of autologous auricular chondrocytes, coculture systems, decellularized auricular extracellular matrices, and 3D printing or bioprinting. Together, these advances indicate that auricular cartilage tissue engineering has moved beyond simple proof-of-concept. However, routine clinical translation remains constrained by unresolved challenges, including long-term shape maintenance, soft-tissue coverage, inflammatory remodeling, scaffold degradation, reproducible manufacturing, regulatory approval, and integration into pediatric reconstructive workflows. At the same time, they reveal a key conceptual limitation: many studies still define success as the formation of “cartilage-like” tissue, even though auricular regeneration ultimately requires a stable elastic organ rather than a histologically acceptable cartilage mass. In this perspective, we argue that the field has reached a stage where conceptual refinement is as important as technical innovation. Future progress, we propose, depends on three related shifts: moving from a generic chondrogenic paradigm to one centered on elastic cartilage biology; redefining scaffolds as instructive microenvironments rather than mere shape-retaining supports; and evaluating translational success not only by feasibility, but also by reproducibility, manufacturability, and long-term clinical robustness. Rather than offering another technique-centered review of auricular reconstruction, this Perspective advances an auricular-specific framework for defining success in elastic organ regeneration. The next major advances, in our view, will come from approaches that integrate elastic cartilage biology, instructive scaffold design, and measurable translational criteria rather than optimizing these dimensions separately.

## 1 Introduction

Auricular reconstruction remains one of the most compelling and visible targets for regenerative medicine (Huang et al., 2023). The external ear occupies a unique position in reconstructive surgery because its clinical value is not merely functional or structural, but also strongly aesthetic, developmental, and psychosocial (Humphries et al., 2022). Patients with microtia, traumatic auricular loss, oncologic defects, or severe deformities often require restoration of a highly recognizable anatomical structure whose success is judged immediately by symmetry, contour, projection, and natural appearance (Yamada et al., 2024; Hellies et al., 2025). This makes the ear both a challenging and an attractive organ for tissue engineering: challenging because its anatomical precision is unforgiving, and attractive because its relatively avascular cartilage core and external location make it more accessible than many internal organs (Khan et al., 2024).

The conventional standard for total auricular reconstruction continues to rely heavily on autologous costal cartilage carving (Brent, 1999; Nagata, 1993; Firmin and Marchac, 2011). This strategy has produced excellent clinical outcomes in experienced hands and remains a mainstay in reconstructive practice (Yamada et al., 2024; Baluch et al., 2014). Nevertheless, its limitations are well known. Rib cartilage harvest is invasive and may be associated with pain, chest wall morbidity, and a substantial operative burden (Brent, 1999; Kim J. et al., 2024). In addition, successful auricular sculpting depends on remarkable technical expertise, which contributes to variability in outcomes across centers and surgeons. The need for multiple operations, the difficulty of reproducing complex fine structures, and the challenge of generating individualized symmetry further underscore the shortcomings of the traditional paradigm (Firmin and Marchac, 2011; Baluch et al., 2014; Ronde et al., 2021).

These limitations explain why auricular tissue engineering has maintained long-standing appeal. Unlike many regenerative targets, the auricle presents a clearly defined geometric goal. Advances in medical image registration and segmentation have expanded the possibilities for image-based anatomical reconstruction and digital modeling (Reddy et al., 2025). Modern imaging can capture the contralateral ear, digital tools can generate mirrored patient-specific designs, and scaffold fabrication technologies can reproduce intricate contours with increasing precision (Zhou et al., 2018; Otto et al., 2021; Dwivedi et al., 2022). Yet geometry alone is not sufficient. A surgically implanted auricular construct must survive implantation, resist deformation, mature into stable tissue, and remain recognizably auricular over time. In other words, the problem is not simply one of fabrication, but one of biological fidelity under clinical conditions.

This distinction is especially important because auricular cartilage is elastic cartilage, not hyaline cartilage. Elastic cartilage differs not only in matrix composition, but in its functional behavior and long-term structural demands. Regeneration of the ear therefore cannot be approached merely as a version of cartilage repair writ large. The central challenge is not to form a generic cartilage matrix, but to reproduce a tissue capable of preserving complex morphology while retaining elasticity and durability under the stresses of implantation, healing, and long-term remodeling.

For this reason, we believe that auricular tissue engineering should now be reframed. The defining question is no longer whether engineered constructs can generate cartilage-like tissue, but whether they can regenerate a living auricular structure that remains biologically elastic, morphologically faithful, and clinically reliable. This conceptual reframing has direct consequences for how the field chooses cell sources, designs scaffolds, defines endpoints, and interprets translational success.

### 1.1 Positioning and novelty of this perspective

Recent reviews have comprehensively summarized microtia reconstruction, autologous auricular reconstruction, scaffold materials, three-dimensional printing, bioprinting, and broader cartilage tissue engineering strategies. These reviews have been highly valuable in describing available techniques and mapping technological progress in the field. However, many of them are primarily organized around reconstructive methods, biomaterial platforms, or fabrication technologies.

The present Perspective has a different purpose. Rather than providing another broad technical overview, we focus on a conceptual and evaluative gap that becomes increasingly important as the field moves toward translation: how should success in auricular cartilage tissue engineering be defined? We argue that auricular engineering should not be judged simply by whether a construct forms cartilage-like matrix, but by whether it regenerates a shape-stable, elastic, and clinically usable auricular structure.

The novelty of this Perspective therefore lies in integrating three dimensions that are often discussed separately: elastic cartilage biology, scaffold-guided morphogenesis, and translational success criteria. By linking these dimensions, we propose an auricular-specific framework that complements previous reviews and helps reorient future studies from proof-of-concept cartilage formation toward clinically meaningful elastic organ regeneration. To make this distinction clearer, Supplementary Table S1 summarizes how the present perspective differs from prior reviews in scope, organizing logic, and translational emphasis.

## 2 Auricular cartilage engineering should target an elastic organ, not generic cartilage

### 2.1 Auricular reconstruction poses a biologically distinct regenerative challenge

One of the reasons auricular tissue engineering has progressed more rapidly than some other craniofacial regenerative applications is the availability of a biologically relevant autologous cell source (Zhou et al., 2018; Melgarejo-Ramírez et al., 2016). In patients with microtia, residual malformed auricular cartilage can often be harvested in small quantities, and this tissue retains key characteristics of elastic cartilage (Melgarejo-Ramírez et al., 2016; Gonzales et al., 2024). From a regenerative standpoint, this is highly advantageous. It means that the starting material already contains cells with lineage memory, matrix preferences, and gene-expression programs related to auricular identity. In principle, this gives auricular tissue engineering a biologically privileged position compared with approaches that depend entirely on nonspecific mesenchymal stem cells or unrelated chondrogenic sources (Zhang et al., 2014; Fisch et al., 2025).

However, this advantage should not obscure the fact that the regenerative target is unusually demanding. The ear is not a flat or mechanically uniform cartilage structure. It is a curved, topographically complex, asymmetric organ with multiple ridges, depressions, transitions in thickness, and exposed margins. Unlike nasal cartilage grafts or simpler cartilage patches, the auricle must maintain an immediately recognizable geometry across its entire surface. Small changes in helix definition, antihelical curvature, lobular relationship, or conchal depth can produce clinically obvious distortions. In this setting, the tolerance for biological contraction, scaffold collapse, or uneven maturation is remarkably low.

Moreover, the clinical environment further complicates regeneration. Auricular constructs are not simply cultured *in vitro* and judged in isolation; they must survive implantation beneath skin or fascial coverage, integrate with surrounding tissues, resist inflammatory remodeling, and maintain their shape under external forces and scar contracture. Thus, a construct that appears promising in histology or small-scale implantation studies may fail when confronted with the full demands of clinical reconstruction.

For these reasons, we argue that auricular cartilage engineering should not be conceptualized as a branch of generic cartilage repair. It is more appropriately understood as elastic organ reconstruction. This distinction matters because it changes the entire logic of study design. If the goal is generic cartilage, then matrix production, proteoglycan content, and type II collagen staining may appear sufficient. If the goal is auricular organ regeneration, then one must ask a more demanding set of questions: does the construct preserve fine-scale contour, generate an elastic matrix, maintain shape over time, resist contraction, and behave like auricular cartilage after implantation? A field that fails to make this distinction risks overestimating progress.

### 2.2 Cartilage formation alone should no longer be considered a sufficient endpoint

Much of the published auricular tissue engineering literature still reflects an older evaluation framework in which the presence of cartilage-like extracellular matrix is treated as the primary marker of success. Histological positivity for proteoglycans, type II collagen, or lacuna-like cellular organization undoubtedly provides useful evidence of chondrogenesis. However, in the auricular context, these parameters are necessary but not sufficient.

The core problem is that many constructs can generate matrix without becoming clinically meaningful auricular tissues. However, representative studies also indicate that histological cartilage formation must be interpreted together with structural and functional outcomes (Zhou et al., 2018; Otto et al., 2021; Gonzales et al., 2024). A construct that shows proteoglycan deposition or type II collagen expression may still be translationally incomplete if it lacks stable three-dimensional contour, elastic fiber organization, mechanical recoil, resistance to contraction, or reproducible behavior after implantation (Fisch et al., 2025; Bernstein et al., 2018; Cohen et al., 2018). Therefore, the key limitation is not the use of histology itself, but the interpretation of histology as a sufficient surrogate for auricular function. Such outcomes are not peripheral technical issues; they strike at the heart of whether a construct can function as a real auricular replacement. In this setting, a narrow focus on histological cartilage formation risks rewarding biological partial success while overlooking translational failure.

This problem becomes even more apparent when considering cell expansion. Because the amount of harvestable auricular cartilage is limited, *ex vivo* expansion is often necessary (Zhou et al., 2018; Bernstein et al., 2018). Historically, expansion has been associated with concern about dedifferentiation, loss of cartilage phenotype, and reduced tissue quality. Yet the more important question is not whether expansion occurs, but whether expanded cells still retain the ability to generate a matrix and structure appropriate for auricular function. Work showing that late-passage auricular chondrocytes can still produce elastin-rich tissue (Fisch et al., 2025; Bernstein et al., 2018) and perichondrium-like organization (Cohen et al., 2018; PubMed, 2026) suggests that the field should move away from simplistic assumptions. Expansion itself is not the endpoint; the preservation of functional auricular identity is.

In our view, auricular regeneration should be evaluated using an auricular-specific success framework. Such a framework would include, at minimum, the following dimensions:

1. Elastic matrix quality, assessed by elastin staining and quantification, elastic fiber organization, collagen II/I ratio, proteoglycan or glycosaminoglycan content, and mechanical recoil testing;
2. Shape stability, assessed by serial three-dimensional scanning, volumetric contraction, contour deviation, preservation of helix and antihelix architecture, and long-term morphometric analysis;
3. Surface and boundary architecture, assessed by perichondrium-like outer-layer formation, superficial cell organization, collagen I-rich boundary structure, and integration with adjacent soft tissues;
4. Implantation robustness, assessed by construct survival, inflammatory remodeling, fibrosis, scaffold degradation, calcification, infection risk, and post-implantation mechanical integrity; and
5. Longitudinal fidelity, assessed by repeated imaging, maintenance of auricular projection and symmetry, durability of elastic recoil, and clinically relevant aesthetic outcomes.

To make this framework more practical, each conceptual criterion should be translated into measurable endpoints. Elastic matrix quality can be assessed by elastin staining, quantitative elastin measurement, elastic fiber organization, collagen II/I ratio, proteoglycan or glycosaminoglycan content, and mechanical recoil testing. Shape stability can be evaluated using serial three-dimensional scanning, volumetric contraction analysis, surface deviation mapping, preservation of helix and antihelix architecture, and long-term contour analysis. Other criteria, including boundary architecture, implantation robustness, and longitudinal fidelity, should likewise be assessed using defined structural, biological, mechanical, and clinical endpoints. These proposed measurable endpoints are summarized in Supplementary Table S2.

A major conceptual task for the field is therefore to redefine what it is willing to call success. We suggest that auricular cartilage engineering should no longer celebrate cartilage formation *per se*, but rather the sustained generation of a tissue that reproduces the structural and biological logic of the native auricle.

**FIGURE 1:**
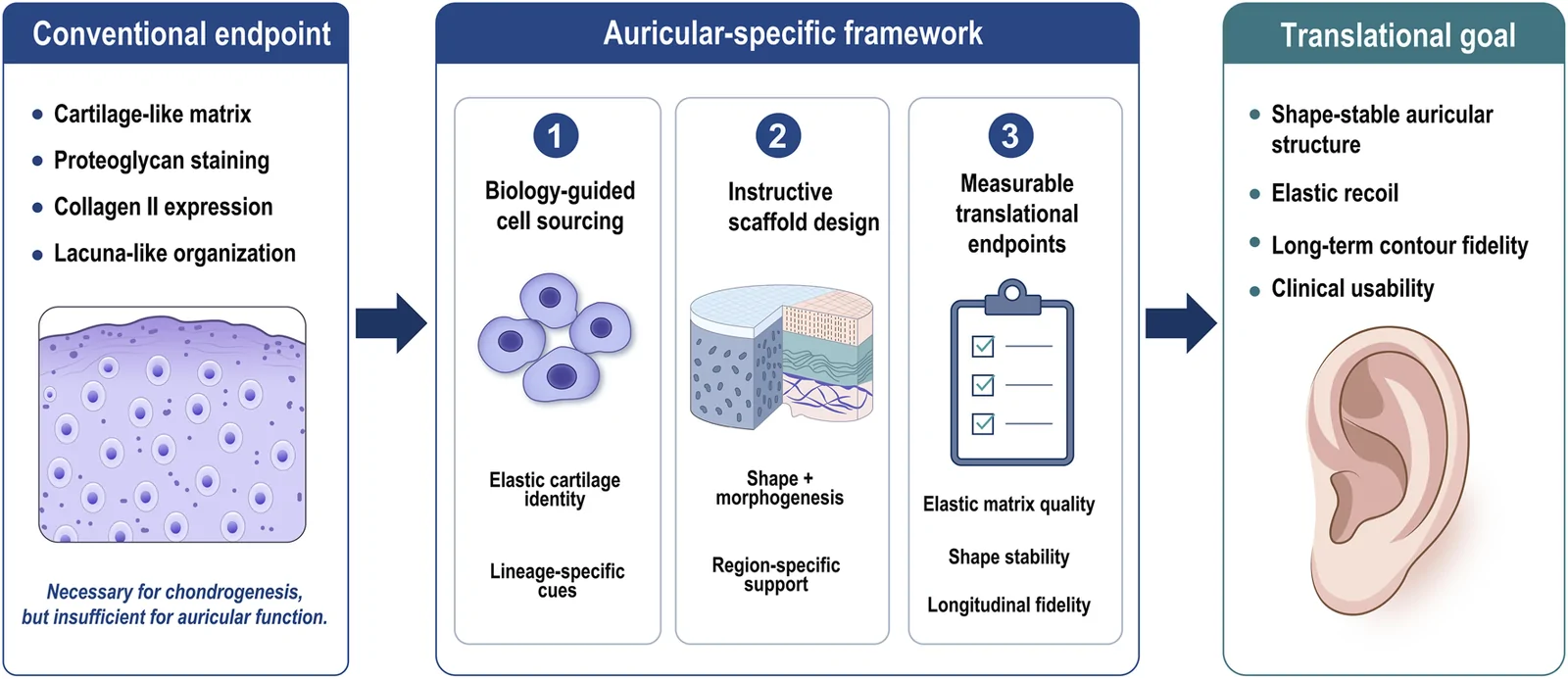
Framework for auricular elastic organ regeneration. Conventional assessment of engineered auricular cartilage has often emphasized cartilage-like tissue formation, including proteoglycan staining, type II collagen expression, and lacuna-like cellular organization (left panel). Although these markers are necessary for confirming chondrogenesis, they are insufficient to predict whether a construct will function as a stable auricular structure. The proposed auricular-specific framework (middle panel) integrates three linked dimensions: biology-guided cell sourcing to preserve elastic cartilage identity, instructive scaffold design to guide morphogenesis and region-specific support, and measurable endpoints to evaluate construct performance over time. Together, these dimensions shift the evaluation goal from simple histological success toward the translational objective (right panel) of generating a shape-stable, elastic auricular organ with long-term contour fidelity and clinical usability.

Taken together, these limitations suggest that auricular cartilage engineering needs a more integrated conceptual framework. The field must move beyond permissive histological endpoints and instead align cell sourcing, scaffold design, and evaluative criteria with the biological and translational requirements of auricular regeneration. This proposed reframing is summarized in Figure 1.

### 2.3 Representative evidence supporting the need for auricular-specific endpoints

A review of representative studies supports the need to evaluate engineered auricular constructs beyond conventional histological markers. Early and recent studies have shown that engineered auricular tissues can demonstrate chondrogenic features, including proteoglycan deposition, type II collagen expression, lacuna-like organization, or elastin-rich matrix formation (Zhou et al., 2018; Otto et al., 2021; Gonzales et al., 2024; Fisch et al., 2025; Bernstein et al., 2018; Cohen et al., 2018). However, the translational relevance of these findings depends on whether such biological maturation is accompanied by preservation of ear-shaped geometry, elastic recoil, mechanical compatibility, resistance to contraction, and reproducibility after implantation. To provide a more structured evidence base for this argument, Supplementary Table S3 summarizes representative studies and highlights how each study supports the need for auricular-specific endpoints beyond conventional histological assessment.

For example, patient-specific ear-shaped cartilage constructs have demonstrated the feasibility of generating clinically relevant auricular tissue using expanded microtia chondrocytes and biodegradable scaffolds, including pilot clinical application (Zhou et al., 2018). However, this type of work also highlights that successful translation requires assessment of aesthetic form, construct stability, individualized geometry, and long-term outcome rather than histology alone. Similarly, biofabricated auricular constructs have advanced the evaluation of shape stability and scaffold architecture, indicating that geometric fidelity and structural persistence should be treated as essential endpoints rather than secondary observations (Otto et al., 2021). Studies using late-passage auricular chondrocytes further show that histological evidence should be interpreted together with biochemical and biomechanical testing, because the central question is not simply whether cells remain chondrogenic, but whether they can generate elastic cartilage with native-like functional properties (Fisch et al., 2025; Bernstein et al., 2018). Coculture-based approaches also illustrate that gross auricular shape, implanted construct behavior, and matrix quality must be evaluated together when assessing the clinical relevance of a cell-source strategy (Fisch et al., 2025; Cohen et al., 2018).

Taken together, these studies do not weaken the value of histological analysis; rather, they show that histology should be embedded within a broader auricular-specific evaluation system. Conventional markers such as proteoglycan staining and type II collagen expression are necessary indicators of chondrogenesis, but they cannot by themselves determine whether an engineered construct will function as a stable elastic auricular structure after implantation.

## 3 Cell sourcing should be biology-guided, not expansion-driven

### 3.1 Auricular chondrocytes remain the biological anchor of the field

Despite intense interest in stem cells and alternative cell sources, auricular chondrocytes remain central to the field for a simple reason: they are the most direct carriers of auricular identity. Their importance lies not only in their ability to synthesize matrix, but also in their lineage-specific biological memory (Melgarejo-Ramírez et al., 2016; Gonzales et al., 2024). These cells have already undergone the developmental programming that makes elastic cartilage distinct from other cartilage subtypes (Yang et al., 2024). As a result, they provide an anchor of biological specificity that is difficult to fully replace with more generic progenitor populations (Melgarejo-Ramírez et al., 2016; Gonzales et al., 2024; Cohen et al., 2018; Yang et al., 2024).

This point is especially important in the context of translational realism. Stem cells may offer scalability, availability, and proliferation advantages, but scalability without specificity is not necessarily helpful in auricular reconstruction. A large number of cells that generate fibrocartilage-like or immature cartilage tissue is less valuable than a smaller population capable of directing truly auricular tissue formation (Fisch et al., 2025; Cohen et al., 2018). For this reason, even when auricular chondrocytes are not used as the sole cell source, they often remain the benchmark against which other strategies are judged.

Auricular chondrocytes are also biologically instructive. Their value is not confined to their direct participation in matrix production. They may shape the surrounding microenvironment through secreted factors, cell–cell signaling, and extracellular vesicle release (Guo and Fan, 2023), thereby influencing the behavior of accompanying cell populations. This suggests that their contribution to regeneration may be broader than simple tissue building; they may help define the molecular context in which auricular cartilage can actually emerge.

Accordingly, we view auricular chondrocytes not merely as one cell option among many, but as the biological reference point of the field. Future strategies may reduce the absolute number of auricular chondrocytes required, but approaches that discard them entirely should be held to a particularly high standard of proof regarding elastic cartilage specificity.

### 3.2 Hybrid and paracrine strategies may offer a more scalable path forward

The principal challenge with auricular chondrocytes, of course, is that they are limited in number. This has driven the development of coculture systems, supportive cell combinations, and paracrine-based strategies intended to amplify regenerative capacity without sacrificing lineage specificity. In many ways, this may represent the most realistic path toward clinically scalable auricular reconstruction.

Coculture systems are conceptually attractive because they distribute biological functions across multiple cell populations (Zhang et al., 2014; Cohen et al., 2018; Landau et al., 2021). Auricular chondrocytes can provide lineage-defining cues, while mesenchymal stromal or stem cells contribute proliferative support, matrix production, immunomodulation, or trophic signaling. In such a framework, auricular chondrocytes do not need to produce all of the tissue directly; instead, they can serve as instructive leaders within a broader regenerative consortium (Cohen et al., 2018; Landau et al., 2021). This approach also aligns with a more modern view of tissue engineering, in which cell populations are valued not only for what they become, but for the signals they provide to one another.

Paracrine strategies represent an extension of this logic. If auricular chondrocytes exert much of their influence by shaping the surrounding microenvironment, then cell-derived extracellular vesicles (Guo and Fan, 2023), conditioned media, or matrix-bound signaling cues may become powerful tools for inducing elastic cartilage-like differentiation in secondary cell populations. Such approaches are especially attractive because they may reduce the need for large quantities of harvested auricular tissue while preserving key aspects of auricular-specific induction.

Nevertheless, hybrid systems introduce their own challenges. They complicate manufacturing, raise questions about compositional consistency, and make mechanistic interpretation more difficult. In addition, not all supportive cells are equally suitable. A scalable cell source that drives matrix formation but promotes fibrotic remodeling, excessive hypertrophy, or loss of elastic properties would be counterproductive. Therefore, the field must resist the temptation to define success in purely volumetric or proliferative terms.

We suggest that future cell-source studies should be organized around the concept of auricular specificity preservation. Instead of asking which system grows fastest or produces the largest tissue mass, investigators should ask which system most reliably reproduces the molecular, structural, and mechanical features of elastic auricular cartilage. This includes evaluating elastin-related pathways, tissue contraction behavior, local matrix architecture, and the ability of constructs to retain shape over time. Put simply, auricular cartilage engineering does not need more cells in the abstract; it needs biologically instructed cells that can sustain auricular identity under clinically meaningful conditions (Gonzales et al., 2024; Fisch et al., 2025; Fisch et al., 2021).

## 4 Scaffolds should evolve from shape-retaining frames to instructive microenvironments

### 4.1 Shape retention is necessary, but no longer sufficient

In auricular engineering, scaffold design was initially dominated by an obvious and legitimate concern: how to preserve ear shape. The geometric complexity of the auricle makes passive tissue formation alone inadequate for reconstruction. Without structural guidance, cell-laden constructs tend to contract, collapse, or lose fine details (Zhou et al., 2018; Otto et al., 2021). As a result, early progress in the field understandably focused on developing molds, frameworks, and biodegradable supports capable of generating recognizable ear-shaped constructs (Cao et al., 1997; Jessop et al., 2016).

This work has been highly valuable. Patient-specific scaffold fabrication based on imaging data, mirrored ear anatomy, and digital modeling has transformed what is technically possible (Zhou et al., 2018; Otto et al., 2021; Kim M. et al., 2024). It is now feasible to generate highly individualized frameworks that reproduce the macroscopic architecture of the ear with a level of precision that would have been difficult to achieve using hand-sculpted approaches alone. This is a genuine translational advance because it directly addresses one of the central limitations of conventional reconstruction: variability in form generation.

Yet it is increasingly clear that shape retention, while essential, is not enough. A scaffold that maintains gross geometry but does not support appropriate tissue maturation will ultimately fail in subtler but equally important ways. If the matrix remains biologically immature, if contraction occurs unevenly across the construct, if the surface layer is unstable, or if degradation is mismatched to tissue formation, then precise initial shaping may not translate into durable clinical success. In this sense, the field has solved part of the geometry problem, but not the full morphogenesis problem.

We therefore argue that shape retention should now be considered a baseline requirement rather than the defining achievement of a scaffold. The more challenging and more important question is whether a scaffold can preserve shape because it successfully guides biological maturation, not merely because it temporarily resists collapse.

### 4.2 The next-generation scaffold should function as a morphogenetic microenvironment

The next-generation of auricular scaffolds should be understood not as inert carriers, but as active morphogenetic microenvironments. A scaffold suitable for auricular regeneration should do more than hold cells in place. It should regulate their spatial distribution, influence their phenotype, shape diffusion gradients, support matrix assembly, reduce unwanted contraction, and ideally help establish a surface organization analogous to native perichondrium. In other words, scaffold design should be integrated with developmental logic rather than treated as a separate engineering problem.

This is where biologically derived materials and hybrid scaffold systems become particularly significant. Decellularized auricular matrices are attractive because they preserve aspects of native extracellular architecture and biochemical composition that synthetic materials may struggle to replicate (Nürnberger et al., 2019; Ziegler et al., 2024; Visscher et al., 2021). Even when their mechanical properties require supplementation, they offer a model for what auricular microenvironmental instruction might look like. Similarly, composite scaffolds that combine structural reinforcement with cell-permissive bioactive compartments may better accommodate the competing demands of contour stability and tissue maturation (Otto et al., 2021; Vernice et al., 2024).

We believe that uniform scaffolds are unlikely to fully meet the needs of auricular reconstruction. The native auricle is not biologically homogeneous, and engineered constructs may need corresponding heterogeneity. Structural zones responsible for contour definition may require greater stiffness or slower degradation, whereas internal regions may prioritize nutrient diffusion and matrix deposition. The outer interface may need to support perichondrium-like organization or interactions with covering soft tissues. This suggests that the future of auricular scaffolds lies not in a single ideal material, but in functionally stratified designs that distribute different regenerative tasks across different regions of the construct (Guo et al., 2025).

This perspective also has implications for bioprinting. The promise of bioprinting does not lie solely in its precision, but in its potential to create controlled heterogeneity. If multiple bioinks, cell populations, and mechanical domains can be arranged in a spatially intelligent manner, then bioprinting may help reproduce not just the outer shape of the ear, but some of its internal organizational logic (Otto et al., 2021; Dwivedi et al., 2022). To realize this potential, however, scaffold design must be informed by auricular biology rather than by manufacturing convenience alone.

### 4.3 Role of perichondrium-like boundary in auricular cartilage regeneration

The perichondrium-like boundary plays a critical role in auricular cartilage regeneration. It not only provides mechanical support to resist contraction and preserve three-dimensional ear shape, but also serves as an instructive interface for chondrocyte orientation and elastic fiber organization. Scaffold designs that promote formation of this superficial fibrous layer may enhance tissue integration, facilitate vascular and nutrient support, and improve long-term stability of engineered auricular constructs. Incorporating perichondrium-like organization as a measurable endpoint can therefore strengthen translational relevance (Fisch et al., 2025; Cohen et al., 2018).

### 4.4 Technical considerations for long-term scaffold degradation

Long-term degradation of scaffolds in auricular cartilage engineering has critical implications for both tissue integration and construct stability (Hu et al., 2025; Zhang et al., 2026). Degradation products, depending on their chemical composition and rate of release, may induce sterile inflammation, trigger fibrous encapsulation (Lyu et al., 2009), and potentially lead to scaffold exposure or extrusion. Such responses can compromise the fine three-dimensional contour of the auricle and interfere with the maturation of the cartilage core. The chemical byproducts may also modulate local immune cell behavior, potentially altering macrophage polarization or fibroblast activity, which in turn affects matrix remodeling and surface stability.

Moreover, improper degradation kinetics or persistent scaffold fragments may increase the risk of secondary infection, particularly when the scaffold is exposed to skin or soft tissue coverage under limited vascularization conditions. These risks underscore the importance of coordinating scaffold degradation with neocartilage formation and mechanical maturation, and of using materials that are biocompatible, immunologically inert, and appropriately cleared after tissue remodeling. Quantitative assessment of degradation rate, local inflammatory response, and integration outcomes should therefore be incorporated as part of the translational success criteria for engineered auricular constructs.

### 4.5 Technical constraints in scaffold design for clinically sized auricular constructs

Translating scaffold concepts into clinically sized auricular constructs requires attention to degradation, mechanics, mass transport, and host–tissue interactions. Scaffold degradation should be synchronized with neocartilage matrix deposition and mechanical maturation. Premature degradation may cause loss of projection, contour blunting, or collapse, whereas delayed degradation may leave persistent foreign material, increase stiffness, impair elastic recoil, or prolong inflammation. Similarly, scaffold mechanics should balance early shape retention with long-term elastic function. Excessive stiffness may preserve the helix, antihelix, and projection but create mechanical mismatch with native elastic cartilage and surrounding soft tissues, whereas overly compliant scaffolds may improve recoil but increase the risk of warping, contraction, or collapse.

Mass transport and implantation biology are equally important in full-sized auricular constructs. Nutrient and oxygen diffusion, waste removal, and cell survival may differ substantially from those in small experimental constructs; therefore, porosity, pore interconnectivity, channel architecture, hydrogel density, and cell distribution require careful optimization. After implantation, calcification, fibrotic encapsulation, chronic inflammation, infection risk, and incomplete integration with skin or fascial coverage may compromise long-term shape and elasticity. These considerations support functionally stratified scaffold designs that coordinate degradation rate, mechanical support, elastic recoil, diffusion, host response, and soft-tissue integration before engineered auricular constructs can be considered translationally robust (Otto et al., 2021; Nürnberger et al., 2019; Ziegler et al., 2024; Visscher et al., 2021; Vernice et al., 2024; Guo et al., 2025).

This requirement is illustrated by several representative studies of clinically sized or patient-specific auricular constructs. Zhou et al. reported the regeneration of patient-specific ear-shaped cartilage using expanded microtia chondrocytes and biodegradable scaffolds (Zhou et al., 2018), followed by its first clinical application in microtia patients. This study demonstrated the translational feasibility of generating individualized ear-shaped cartilage, but also underscored that clinical success depends on aesthetic shape preservation, construct stability, and long-term follow-up rather than cartilage formation alone. Similarly, Otto et al. developed a shape-stable auricular construct using hybrid biofabrication and scaffold-based design, highlighting that full auricular geometry requires coordinated control of scaffold architecture, cell distribution, neocartilage formation, and mechanical support (Otto et al., 2021). More recently, Vernice et al. engineered full-scale auricles using decellularized cartilage xenograft within a three-dimensional printed external scaffold that mimicked the size, shape, and biomechanical properties of the native human auricle (Vernice et al., 2024). Although this approach provided an important full-scale preclinical example, the authors also noted the need for longer-term studies and improved degradation behavior before clinical translation. Together, these examples show that clinically sized auricular constructs are limited not by a single factor, but by the coordination of scaffold degradation, mechanical persistence, tissue maturation, host response, and long-term shape fidelity. Therefore, comparative evaluation of successful and incomplete full-scale constructs should become an essential part of future auricular scaffold studies.

## 5 Discussion

### 5.1 Translational promise and unresolved barriers

Auricular cartilage tissue engineering is no longer defined only by conceptual promise. Patient-specific ear-shaped constructs, expanded auricular chondrocytes, coculture systems, decellularized matrices, and advanced scaffold fabrication have collectively demonstrated that the field has moved beyond simple proof-of-concept cartilage formation (Zhou et al., 2018; Otto et al., 2021; Zhang et al., 2014; Fisch et al., 2025; Bernstein et al., 2018; Cohen et al., 2018). These advances justify cautious translational optimism, but they should not be interpreted as evidence that clinically routine auricular regeneration is imminent (Yamada et al., 2024; Hellies et al., 2025).

Several major barriers remain unresolved. Long-term shape maintenance remains a central challenge, because even small degrees of contraction, warping, contour blunting, or loss of projection can compromise the aesthetic and reconstructive value of an auricular construct (Ronde et al., 2021; Zhou et al., 2018; Otto et al., 2021). Implantation beneath clinically relevant soft-tissue coverage also introduces additional biological variables, including nutrient diffusion, vascularized fascial or skin coverage, wound healing, fibrosis, infection risk, and inflammatory remodeling. In parallel, scaffold degradation must be coordinated with matrix deposition and mechanical maturation; otherwise, premature degradation, persistent stiffness, calcification, or mechanical mismatch may impair long-term function (Otto et al., 2021; Nürnberger et al., 2019; Ziegler et al., 2024; Visscher et al., 2021). Beyond these biological and material challenges, reproducibility, good manufacturing practice (GMP)-compatible manufacturing, sterility assurance, regulatory approval, cost, surgical workflow, and integration into pediatric treatment timelines must be addressed before engineered auricular constructs can be widely adopted (Yamada et al., 2024; Hellies et al., 2025; Zhou et al., 2018; Guo et al., 2025).

Therefore, we use the concept of a translational transition cautiously. The field is not yet at the stage of routine clinical implementation; rather, it is entering a phase in which promising experimental and early translational studies must be tested against stricter criteria of durability, reproducibility, manufacturability, safety, and clinical workflow compatibility. This more cautious interpretation reinforces the central argument of this Perspective: auricular tissue engineering should be evaluated not only by whether cartilage-like tissue can be produced, but by whether a stable, elastic, reproducible, and clinically usable auricular structure can be generated and maintained over time.

### 5.2 Future progress will depend on redefining what counts as success

Our central argument is straightforward: auricular tissue engineering should stop asking whether it can make cartilage, and start asking whether it can regenerate an auricle that remains recognizably auricular over time. This shift in wording may appear subtle, but it has substantial practical consequences. First, it changes what should be measured. Investigators should prioritize long-term shape fidelity, elastic matrix organization, perichondrium-like boundary formation, and resistance to contraction (Gonzales et al., 2024; Fisch et al., 2025; Cohen et al., 2018; PubMed, 2026), rather than relying predominantly on short-term cartilage histology. Second, it changes what should be optimized. Instead of maximizing isolated variables such as cell yield or scaffold printability, researchers should optimize integrated systems that preserve auricular identity across the full lifecycle of the construct. Third, it changes what should count as translational readiness. Constructs should be judged not only by laboratory performance, but also by reproducibility, manufacturing feasibility, quality control compatibility, and their capacity to withstand clinically realistic implantation conditions (Ronde et al., 2021; Zhou et al., 2018; Vernice et al., 2024). In our view, future progress in the field will depend on three converging priorities. The first is auricular-specific biology: understanding how elastic cartilage differs from other chondral tissues in matrix assembly, developmental signaling, and long-term maintenance. The second is instructional system design: combining cells, scaffolds, and signals in ways that guide tissue formation rather than merely permitting it. The third is translational discipline: building constructs and protocols that can move beyond proof-of-concept into standardized, reproducible clinical pathways. If these priorities are pursued together, auricular cartilage engineering may emerge as one of the clearest examples of how regenerative medicine transitions from tissue fabrication to organ-informed reconstruction. If they are pursued separately, the field may continue to generate impressive constructs without fully crossing the threshold into dependable clinical practice.

## Data Availability

The original contributions presented in the study are included in the article/Supplementary Material, further inquiries can be directed to the corresponding author.
