# Correction: Auricular cartilage tissue engineering: from making cartilage to regenerating a shape-stable elastic organ

**DOI:** 10.3389/fbioe.2026.1974908

**Published:** 2026-09-01

**Authors:** Hongyu Zhang, Jingwei Feng, Jiajun Zhi, Yiwen Deng, Tianqi Yu, Haiyue Jiang, Jiaxian Lin

**Affiliations:** 1 Plastic Surgery Hospital, Chinese Academy of Medical Sciences and Peking Union Medical College, Beijing, China; 2 The Affiliated Nanhua Hospital (Nanhua Clinical College), University of South China, Hengyang, Hunan, China

**Keywords:** 3D printing, auricular cartilage, chondrocytes, elastic cartilage, microtia, regenerative medicine, scaffold, tissue engineering

Affiliation 2 “The Affiliated Nanhua Hospital (Nanhua Clinical College), University of South China, Hengyang, Hunan, China” was omitted from the paper. This affiliation has now been added for author Jiaxian Lin.

The original version of this article has been updated.

